# SYNDEEP: a deep learning approach for the prediction of cancer drugs synergy

**DOI:** 10.1038/s41598-023-33271-3

**Published:** 2023-04-15

**Authors:** Anna Torkamannia, Yadollah Omidi, Reza Ferdousi

**Affiliations:** 1 Department of Health Information Technology, School of Management and Medical Informatics, Tabriz University of Medical Sciences, Tabriz, 51656/65811 Iran; 2 Department of Pharmaceutical Sciences, College of Pharmacy, Nova Southeastern University, Fort Lauderdale, FL 33328 USA

**Keywords:** Computational biology and bioinformatics, Computational models

## Abstract

Drug combinations can be the prime strategy for increasing the initial treatment options in cancer therapy. However, identifying the combinations through experimental approaches is very laborious and costly. Notably, in vitro and/or in vivo examination of all the possible combinations might not be plausible. This study presented a novel computational approach to predicting synergistic drug combinations. Specifically, the deep neural network-based binary classification was utilized to develop the model. Various physicochemical, genomic, protein–protein interaction and protein-metabolite interaction information were used to predict the synergy effects of the combinations of different drugs. The performance of the constructed model was compared with shallow neural network (SNN), k-nearest neighbors (KNN), random forest (RF), support vector machines (SVMs), and gradient boosting classifiers (GBC). Based on our findings, the proposed deep neural network model was found to be capable of predicting synergistic drug combinations with high accuracy. The prediction accuracy and AUC metrics for this model were 92.21% and 97.32% in tenfold cross-validation. According to the results, the integration of different types of physicochemical and genomics features leads to more accurate prediction of synergy in cancer drugs.

## Introduction

Cancer is one of the most detrimental diseases with high mortality worldwide and is considered a challenging barrier in terms of increasing life expectancy^1^. The currently used treatments fail to satisfactorily cure the disease, in large part due to the emergence of drug resistance, and severe side effects^2,3^. In cancer therapy, the foremost target is usually specified in eradicating malignant cells using anticancer cytotoxic drugs through the induction of apoptosis and cell death in the diseased cells and tissues. However, cancer cells can develop escape mechanisms, initiate bypasses in the networks, and emerge alternative pathways for further proliferation and recurrence^4,5^. Remarkably, combinational pharmacotherapy might be the prime strategy that can intensify the therapeutic impacts of anticancer drugs and overcome the drug resistance mechanisms of cancer cells^6,7^. Such an approach can impose synergistic effects and potentially reduce the dose of monotherapy and drug resistance and avoid toxicity while the efficacy of the drug increases^8,9^. Hence, the discovery of the drug combination with synergistic effects is of eminent necessity in treating cancer.

Combinational pharmacotherapy should be provided and designed by in vitro and in vivo experiments based on the US Food and Drug Administration (FDA), the European Medicines Agency, and the World Health Organization guidelines^10^. It should be noted that predicting the possible drug combinations with synergistic effects solely via in vitro and/or in vivo experimentation is an extremely laborious task with no/trivial outcomes^11^. Besides, predicting the synergistic drug combination with clinical experiments is inefficient, time-consuming, and cost-intensive^7,12^. Therefore, in silico approaches can be reliable tools to facilitate and prioritize identifying synergistic drug candidates for experimental strategies^13^.

The use of the in silico approaches as powerful tools has empowered the opportunity of exploring the wide variety of synergistic gaps with the diversity of chemical structures of drugs and genomic data from cancer cell lines^9,14^. Such approaches have paved the way for clinical trial experiments by providing accurate and adequate predictions^15^. Accordingly, over the last years, predicting drug combinations with synergistic effects has been increased by computational methods and has provided satisfactory outcomes. In this line, *Jiang *et al*.* developed a computational model to predict synergistic drug combinations on 39 cell lines using a graph convolutional network^16^. They used the multimodal network of the drug-drug synergy network, drug-target interaction network, and protein–protein interaction network. As a result of their analysis, the value of AUC was higher than 80%. The AuDNNsynergy method was proposed by *Zhang *et al*.*^17^, which identified the synergistic effects based on chemical structure and genomic data. As a result of this approach, the omics data enhance prediction accuracy, and the value of Pearson correlation obtained 0.74. DeepSynergy was developed to detect the drug combinations effect based on the deep neural network^18^. The method has utilized the chemical descriptors of drug pairs and gene expression profiles. DeepSynergy demonstrated the best performance compared to other state-of-art methods. *Yang* and colleagues proposed a model based on the functional similarity of target proteins to prioritize and stratify synergistic drug combinations^19^. This approach capitalized on the protein targets to predict synergy effects on breast cancer cell lines and experimentally validated the BRAF/insulin receptor combination in 48 colorectal cancer cell lines.

In the current study, a novel deep neural network model was proposed as an SYNDEEP to predict the synergistic drug combinations based on cancer cell lines and drug information. The model utilized the different features of physicochemical, genomic, protein–protein interaction, and protein-metabolite interaction information. Then the feature network was constructed based on eight various feature groups. The vector of features was developed according to the structure of the feature network. Finally, the feature vectors are fed into the deep neural network to achieve synergistic prediction results.

## Results

This section summarizes the results of the synergy prediction model on the NCI-ALMANAC dataset. The 74 drugs were utilized in this study. The final experiment dataset consists of 22,228 drug pairs combinations of 74 unique drugs against 60 cell lines. Also, the final features for one drug consisted of 777 features, and total number of the features for a pairs of drugs were 1614 with similarity measurement and cell lines. In addition, the diverse hyperparameters of the algorithms were implemented to adjust the optimal state. As a result, the final optimization parameters are as follows: SNN: hidden layer = 1, dropout rate = 0.8, epochs = 300, learning rate = 10^−2^, KNN: n = 4, metric = 'Minkowski', p = 2, SVM: degree = 3, kernel = ’linear’, cache size = 200; RF: max depth = 4, n estimators = 10; GBCs: max depth = 3, n_estimators = 100.

### Performance of deep neural network with different feature groups

SYNDEEP was implemented with different feature group to evaluate performance. Furthermore, to confirm the deep neural network is robust to training, we implemented tenfold cross-validation (CV) for all six feature group groups. Table 1 summarizes the performance results of six groups. The results highlighted that the four groups achieved accuracy over 90%, and the two remaining groups achieved accuracy of 89%. Best outperforming score was achieved by DC_networkII with the highest accuracy of 92.21%, and the second highest accuracy was achieved by DC_networkI with 92.16%. By including the protein–protein interaction and protein-metabolite interaction similarity score on DC_networkI to generate DC_networkII, the accuracy score slightly increased.

**Table 1:** The overall results of the performance on six groups of features.

Group name	ACCU. (%)	Sen. (%)	Spec. (%)	Prec. (%)	F_score_ (%)	MCC. (%)	AUC (%)	Kappa (%)
DT_CL	90.08	89.86	90.29	90.23	90.05	80.16	96.14	80.16
DCGM	89.78	87.84	91.37	91.38	89.57	79.63	95.80	79.57
DCGME	89.63	87.03	92.22	91.78	89.34	79.36	95.94	79.26
DCGMEM	90.21	89.32	91.10	90.92	90.12	80.44	95.96	80.43
DC_networkI	92.16	91.31	93.00	92.88	92.09	84.33	97.14	84.32
DC_networkII	92.21	91.53	92.91	92.80	92.15	84.41	97.32	84.37

It can be seen from the results that by adding gene mutation and gene expression information to the DT_CL group, accuracy slightly dropped. However, accuracy increased significantly when combined with other feature groups. To further investigate the effect of gene mutation and gene expression on synergy prediction, we left out their information. Substantial differences were not observed in the forecast's performance by excluding the information (*ACCU*. = 92.21%). In drug investigations, gene mutations and gene expression are highly predictive^20^. In this sense, adding their information seems crucial to predicting the synergy effect.

The DC_networkII feature set group was selected as a complete feature set group. To better investigate the model’s prediction ability, we obtained the values of sensitivity, specificity, precision, F_-score_, Matthews correlation coefficient (MCC), AUC, and Cohen’s kappa. The considered evaluation metrics comprehensively reflect the model performance in the values of F_-score_, MCC, and AUC were obtained 92.15%, 84.41% and 97.32%, respectively. In addition, the Kappa value represents the model's inter-rater reliability with a score of 84.37%.

**Figure 1:**
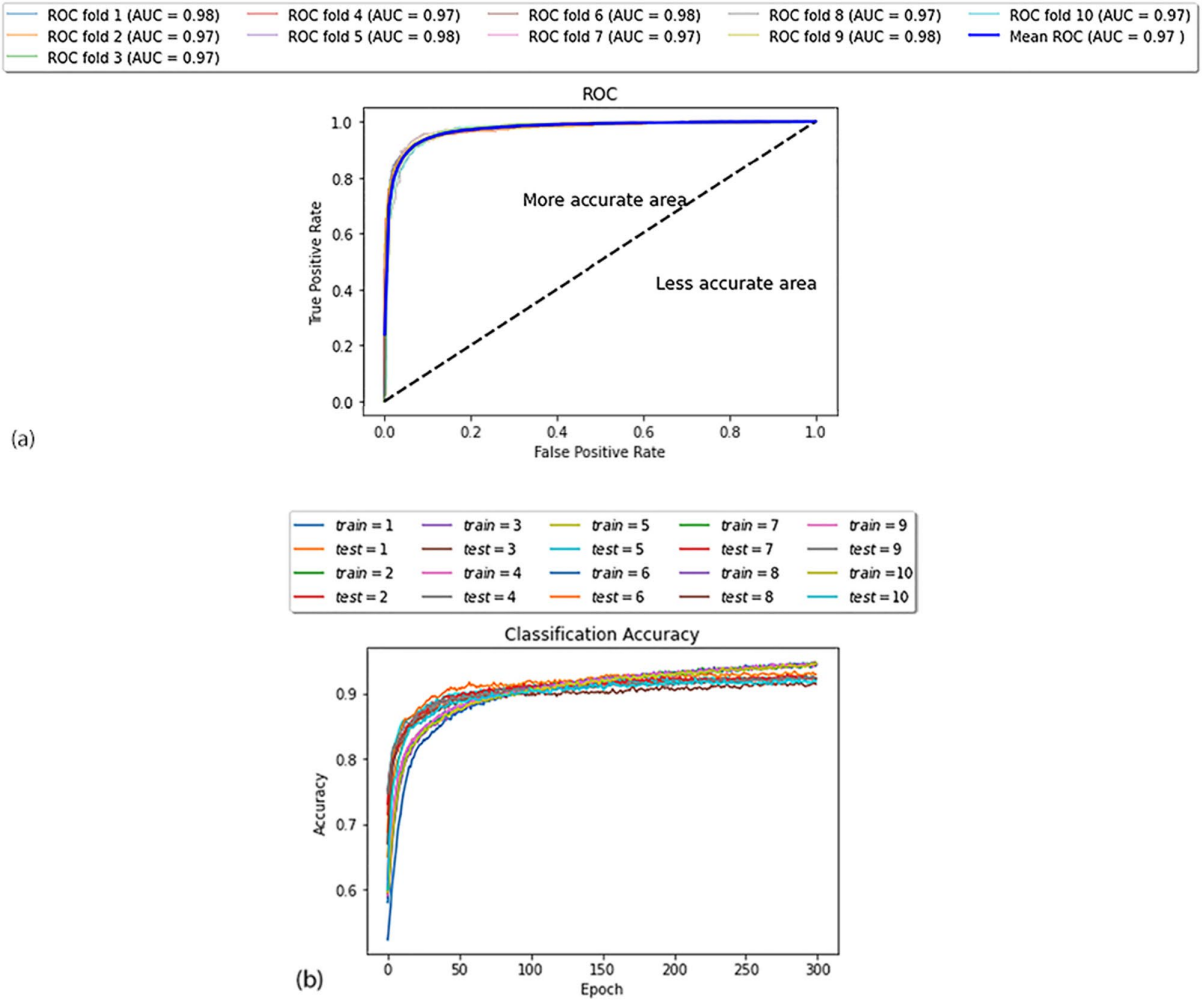
The ROC and accuracy diagram of tenfold-cross validation. (**a**) The ROC curves and (**b**) accuracy diagram were generated on the entire feature set.

Figure 1 shows the area under the receiver operating characteristic curve (ROC-AUC) and the accuracy in evaluating the performance of each fold. As can be seen from the graphs, the curves obtained by ten folds had the most covering coordinate space. The result of the tenfold cross-validation indicates that SYNDEEP provided a more reliable performance on the entire feature set.

### Performance comparison of the various machine learning methods

We reported the primary evaluation criteria for SYNDEEP, SNN, KNN, SVM, RF, and GBCs in Table 2. We observed from the table that SYNDEEP achieved the highest results in the entire evaluation criteria compared to other methods. The three models with the highest performance based on accuracy were (i) SYNDEEP, (ii) the GBCs, and (iii) the KNN. Accordingly, SYNDEEP accuracy was 92.21%, which is approximately 3.8% and 4.23% higher than the GBCs model, and the KNN, respectively. Among the models examined, the RF achieved poor accuracy (74.85%). Also, SYNDEEP achieved remarkable results in Matthews Correlation Coefficient (*MCC*. = 84.41%), representing the correlation between predictions and labels. Notably, SYNDEEP obtained a high Kappa score (*Kappa*. = 84.37%) while the RF had the lowest score (*Kappa*. = 49.69%).

**Table 2:** The overall results of the different state-of-art methods.

Method	ACCU. (%)	Sen. (%)	Spec. (%)	Prec. (%)	F_score_ (%)	MCC. (%)	AUC (%)	Kappa (%)
Gradient boosting classifiers	88.41	88.73	88.10	87.97	88.35	76.83	0.94	76.83
SYNDEEP	92.21	91.53	92.91	92.80	92.15	84.41	0.97	84.37
Random forest	74.85	80.15	71.15	66.00	72.39	50.49	0.84	49.69
Support vector machines	84.23	84.42	84.04	83.92	84.17	68.46	0.90	68.46
Shallow neural network	86.61	86.48	86.74	86.71	86.59	73.23	0.93	73.23
k-nearest neighbor	87.98	84.35	92.36	93	88.47	76.39	0.88	75.99

**Figure 2:**
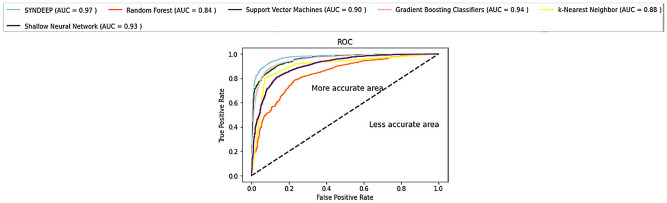
ROC curve comparison of different machine learning methods.

The ROC curve was used as another evaluation measure. The ROC curve plots the true-positive rate (TPR) versus the false-positive rate (FPR). The area under the ROC curve (AUC) was calculated to reflect predictive accuracy. Figure 2 shows the visualization of the area under the ROC curve for SYNDEEP and the other classifiers to evaluate the performance of binary predictions. The AUCs for SYNDEEP, GBCs, and SNN were 0.97, 0.94 and 0.93, respectively. This shows SYNDEEP's potential for synergy prediction in drug combinations.

We conducted McNemar's Test to assess classifiers' performance. The null hypothesis of this test states that the probability of the synergistic effect being correctly identified is equal to the probability of the non-synergy effect being correctly identified. Also, the probability of the synergy effect being incorrectly classified is equal to the probability of the non-synergy effect being incorrectly classified. The p-value is calculated, and a p-value < 0.05 is considered significant, thus rejecting the null hypothesis. Hence, we obtained X^2^ = 154.0, with a p-value of 0.0, which is below the set significance threshold (p-value = 0.05, degree of freedom = 1) and leads to rejection of the null hypothesis; we can conclude that SYNDEEP’s performance is reliable. The values of McNemar’s Test for GBC, RF, SVM, SNN, and KNN were achieved at 248.0, 363.0, 344.0, 295.0, and 154.0, respectively. Furthermore, RF and KNN obtained p-value < 0.05, while the comparison between GBC, SVM, and SNN gave a non-significant p-value > 0.05, so the null hypothesis was accepted. Generally, SYNDEEP achieved substantial performance compared with other methods to predict drug synergy.

## Discussion

Cancer remains the primary cause of morbidity and mortality worldwide, despite the pharmaceutical and clinical research in cancer treatment^21^. Furthermore, the mono-therapeutic strategies are inefficient in cancer treatment because the targets are single proteins or pathways, and drug resistance occurs in these strategies^22,23^. Accordingly, combining pharmacotherapy with synergistic effects is a promising approach^6,7^. However, it is infeasible to identify and examine the possible combinations of anticancer drugs^24^. Therefore, in silico approaches are beneficial to overcome the limitations.

Hence, we have developed a novel deep neural network model as an SYNDEEP that accurately predicts the synergistic effect of drug combinations for cancer cell lines. In this study, a data-driven approach to predict the synergy effects of drug combinations.

Previous studies used a small subset of genomic and drug-related data to predict synergy^16–19,25,26^. Some studies, such as Jiang’s model^16^, have considered drug-drug synergy, drug-target interaction, and protein–protein interaction networks based on 39 cell lines. Moreover, the DeepSynergy^18^ model has been defined based on chemical descriptors and genomic features on 39 cell lines. AuDNNsynergy^17^ is another drug synergy model that uses multi-omic and chemical data for synergy prediction. The most striking result among previous studies emerged from the studies that utilized genomic and drug information.

To the best of our knowledge, there is no study in the literature that utilized the comprehensive feature set of genomic, physicochemical, and drug information to predict synergy. Utilizing genomic, PPI, PMI, and physicochemical data is important for overcoming drug resistance. The genomic data would be practical for predicting the drug combination synergy^27–29^. In this study, a novel comprehensive feature set of genomic, physicochemical, and drug information (i.e. drug-target, protein–protein interaction, protein-metabolite interaction, gene mutation, gene expression, differential methylation, chemical structure, and cell lines) was constructed. Therefore, we used the comprehensive feature set of various data types to optimize the model’s performance.

The modification in depth and width of model architecture is the pivotal factor in deep neural networks to enhance performance^30^. Generally, applying this property of deep neural networks leads to developing highly impactful architectures for diverse tasks^30,31^. Several previous studies have utilized network-based and machine-learning methods, such as random forests and extremely randomized trees^14,25,32–34^. In contrast, rare studies used deep neural networks that focused on a small range of features^18,35–40^. Thus, we have used comprehensive biological features with a deep neural network. This strategy obtained satisfactory results, as shown in Table 2.

The Kappa value of SYNDEEP was 84.37%. The Kappa value demonstrated that classes have independent distributions. Few related studies in the literature have calculated the kappa coefficient^16–18^, while most studies only reported the accuracy/ AUC value^14,34,37,38^. The kappa values of DeepSynergy, AuDNNsynergy, and Jiang’s model were 0.51, 0.51, and 0.584, respectively. The kappa value in previous studies was just over 0.50%^16–18^, which may represent fair to a reasonable agreement beyond chance.

Previous studies used different datasets to predict the drug combination effects based on the deep neural network^17,39–41^. The NCI-ALMANAC and Merck datasets are the two large-scale pan-cancer datasets employed in most drug synergy prediction studies. For example, among these studies, the performance of the DeepSynergy, AuDNNsynergy, TranSynergy^39^, and Jiang’s models has been evaluated on the Merck dataset. The nature of these studies in terms of recruited information (e.g. multi-omics, phenotypic and biophysical features) was different. For example, some studies utilized drug pathways information, gene expression information, and/or microRNA information. Several previous studies reported the accuracy value, some of which stated the MSE value^17,18,39–41^. The accuracy values of DeepSynergy, AuDNNsynergy, and Jiang’s models were 0.92%, 0.93% and 0.919%, respectively.

In this study, SYNDEEP's performance has been tested on the NCI-ALMANAC dataset. Moreover, among the previous studies, the SYNPRED^40^ and *Xia *et al.^35^ utilized the NCI-ALMANAC dataset based on a deep neural network. The accuracy value of SYNPRED was 0.85%, while Xia’s model^35^ reported the R^2^, Pearson correlation, and Spearman correlation values.

In this study, the accuracy value of the novel deep neural network was 92.21%. While the AuDNNsynergy has reported a value score of 0.93% but the kappa value was 0.51%. However, the results of the current study highlighted the strength of the presented SYNDEEP, which was the large volume of features and network of features. Hence, the proposed model achieved a high score in kappa and accuracy values *(Kappa*. = 84.37%, *ACCU.* = 92.21%).

Evidence of in silico methods represented that combining the biological, chemical, and phenotypic properties had a substantial role in the modeling and prediction procedures^42,43^. According to the performance result of feature sets, it was evident that combining the genomic and physicochemical features had significant effects on improving the performance.

The network-based approaches evaluate the interactions among the various agents. Agents in the prediction of drug effects have different natures. Several studies have used network-based analysis to predict the drug combinations effect^19,25,26,34,37^.

Previous studies prove that integrating the genomic data, drug targeting networks, chemical structure, cell lines, genomic profiling data, gene mutation, gene expression, and protein interaction based on the network approach has a significant role in synergy prediction^19,25,26,34,37^.

Therefore, we have investigated the effect of drug interactions among the diverse feature agents (i.e. drug-target, protein–protein interaction, metabolite-protein interaction, gene expressions, gene mutations, chemical structures, cell lines, and differential methylation). However, the previous studies investigated and analyzed the network of interaction while not examining the network by deep neural network^19,25,26,34,37^.

Here, we have used a network of features comprised of different relationships based on the nature of the information for the first time. As shown in Table 2, the features network has achieved high performance and improved the deep neural network model.

Table 2 has proved that deep neural networks had superior outcomes compared to other state-of-art methods by the network of features.

Deep neural networks have significant capabilities in complex dimensional spaces by various structures^44,45^. Therefore, deep neural network algorithms have been proven to be a more capable system and have a better accuracy rate than other algorithms in classifying synergistic effects in drug combinations.

## Conclusion

The current study proposes SYNDEEP for the prediction of synergy in drugs combination. It is well known that the critical factor in predicting drugs effect is extracting practical features, so the main superiority of this study was the network of extensive features. SYNDEEP integrated the various type of physicochemical and genomics information to generate the comprehensive features set for successfully and robustly predicting drug combinations’ effects. SYNDEEP obtained 92.21% prediction accuracy utilizing the tenfold cross-validation in the NCI-ALMANAC dataset. In the experiment, we compared SYNDEEP, SNN, KNN, SVM, RF, and GBC methods that SYNDEEP achieved superior performance. The result indicated that the deep neural network is the competent learning technique to determine synergy. For better and more accurate predictions in the future, updating and complementing the information of cell lines and targets of drugs are needed. Although the *in silico* approaches provide substantial insights into in vivo and in vitro experiments and accelerate the procedures, well-designed experimental investigations are required to prove the data resulting from *in silico* computational analyses.

## Materials and methods

**Figure 3:**
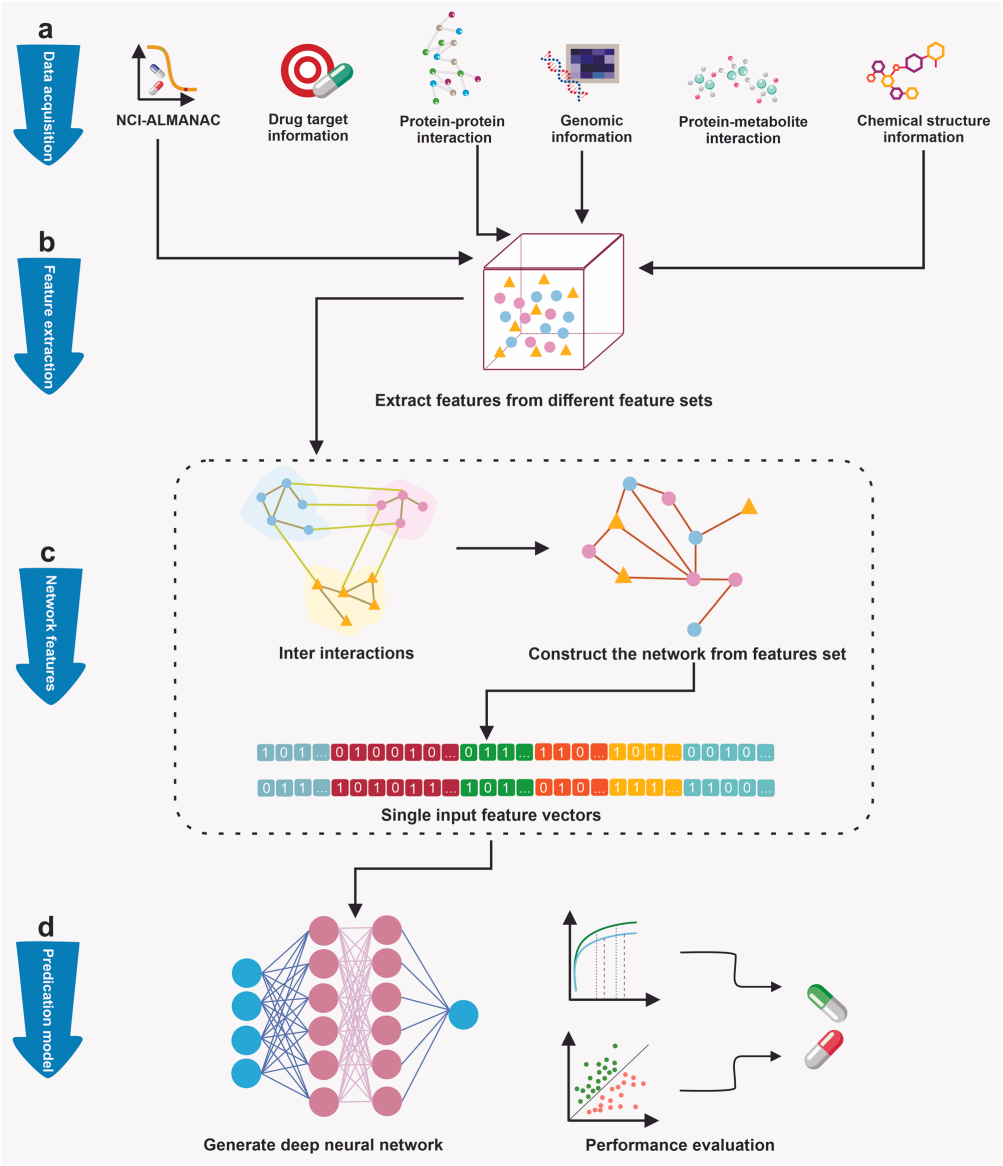
Overall steps to construct drug synergy prediction model. (**a**) eight feature sets were used to predict the synergy effect, drug-target, protein–protein interaction, protein-metabolite interaction, genomic features (gene expression, mutations, and differential methylation), chemical structure, and cell lines. (**b**) Features from different sets were extracted to generate the network. (**c**) The heterogeneous network was created from various features. (**d**) The prediction model was constructed to predict drug combinations’ effects.

The first step of the proposed deep neural network was data preparation which consisted of data acquisition, features extraction, and network of features construction. The next step was prediction model construction which comprised the synergy prediction and model evaluation. Figure 3 illustrates a schematic representation of how the model uses the distinct features to predict synergistic drug combinations. The network of features is recruited as input for a deep neural network to predict the synergistic effect. The network has been constructed from the sequence of physicochemical, genomic, protein–protein interaction, and protein-metabolite interaction data. The data has been produced from different databases based on drug-pairs information. The following figure illustrates the overall steps of the study.

## Data acquisition

In this study, the NCI-ALMANAC dataset was used^46^. This dataset is the most well-known anticancer drug combinations effect dataset, covering the combinations of drug pairs against the NCI-60 cell lines with different concentrations. The drugs in the dataset contain FDA-approved drugs in oncology. The NCI-60 panel comprises 60 human tumor cell lines derived from nine various tumor types. The NCI-ALMANAC introduced the ComboScore to quantify the combination benefit of pairs of drugs which initially modified the version of the Bliss independence score. The ComboScore defines the combination activity by three classes of positive, negative, and zero values. Hence, the combination of drug pairs with positive values represents to be synergistic, whereas the negative values indicate antagonist combinations and zero values correspond to additive combinations.

The ComboScore has defined the score range from 1 to 200 as synergistic, and − 1 to − 228 has specified the antagonistic effect. The score of drug combinations was converted to 1 and 0 to reduce the computational complexity. One represents the synergistic drug-pairs combination, and 0 illustrates the antagonistic drug-pairs combination. By sorting the dataset, the number of antagonist combination effects was more than the synergistic combinations. To avoid the occurrence of an imbalanced dataset, we selected an equal number of drug pairs based on the number of synergistic combinations.

In the present study, different feature groups have been extracted for each drug in the dataset. The extracted feature groups are as follows:

### Extraction of the drug-target interactions

The total indexed drugs and related information in DrugBank(version 5.1.8.) was downloaded^47^. In the next step, the FDA-approved anticancer drugs were extracted from the XML file of DrugBank. Hence, the list of drug-target interactions (DT) for each anticancer drug based on protein targets was elicited. For each drug, the pairs of (Di, DTj) were created, where D_i_ was observed in the approved drug list, and DT_j_ was extracted as a protein target.

### Extraction of the protein–protein interactions

The critical resource of protein–protein interaction data is the STRING (Search Tool for the Retrieval of Interacting Genes/Proteins)^48^ database which is a reliable tool for providing the properties of proteins. As the first step, the sequences of proteins and accessions were retrieved from STRING (version 11.5). Then, protein–protein interaction (PPI) was extracted based on every protein related to DTs. Next, the pair of DTs and PPI was created as (DT_i_ and PPI_j_), where DT_i_ $\in${protein target reported for drug_i_} and PPI_j_ $\in${PPI stated for protein target_j_}.

### Extraction of the genomic features (gene expression, mutations, and differential methylation)

The Catalogue Of Somatic Mutations In Cancer (COSMIC)^49^ database represents worthwhile information on genomic sequences and microarray expression data. The COSMIC database provides extensive data for individual genes/ cell lines. The COSMIC (version 95-24) has provided the individual files for gene expression (GE), mutation (GM), and differential methylation (DM), which all relevant files downloaded. However, the vast majority of information was not relevant to predicting synergy, which might result in a bias. Hence, we filtered the data based on the cell lines and protein targets. As a result, we had the pairs of (D_i_, GE_j_), (D_i_, GM_k_), and (D_i_, DM_n_), where D_i_ was considered in the list of drugs, GE_j,_ GM_k_ and DM_n_ were extracted as gene expression, mutation, and differential methylation.

### Extraction of the protein-metabolite interaction

The Human Metabolome Database (HMDB)^50^ was utilized to construct the protein-metabolite interaction(PMI) data in this study. The metabolite and protein data lists were downloaded from HMDB version 4.0. The list of the corresponding metabolites for every protein in DTs was extracted. Then, the pair of (DT_i_, M_j_) was considered, where DT_i_ $\in${protein target reported for drug_i_} and M_i_ $\in${metabolite reported for protein_j_}.

### Extraction of the chemical structure

We used the Morgan fingerprint counts, total polar surface area, molecular weight, logP, aliphatic and aromatic rings, H-bond donors, and acceptors as chemical structures (CF). The Morgan fingerprint counts are designed to represent the number of times a particular substructure detects in the molecule. To integrate the features into the deep neural network model, we used the RDKit library. As a result, chemical features for each drug were obtained.

### Dimension reduction based on the similarity measure

In total, there were 19,888 features for one drug except for cell lines. The entire features for drug pairs have consisted of 39,776 features with cell lines. These extensive features are considered the high dimensionality problem in constructing the synergy prediction model. The PPI and PMI comprised the high-dimension features among the other feature groups. Hence, the Russell-Rao similarity measure^51^ on PPI and PMI on pairs of drugs was used to overcome this issue. Therefore, the similarity values of PPI and PMI were included in the vector instead of the total features. The Russell-Rao similarity measure is defined as follows:

$${\mathrm{S}}_{\mathrm{Russell}-\mathrm{Rao}}=\frac{x}{d}$$

where *x* denotes the number of features where the values of vector one and vector two are one, which means positive matches, *d* denotes the total sum of the length of vectors.

### Feature network construction

The network feature in SYNDEEP (Fig. 3) was a heterogeneous network consisting of eight components: DT, PPI, PMI, GM, GE, DM, CF, and cell line (CL). In the previous section, it was described how to build each group of features.

**Figure 4:**
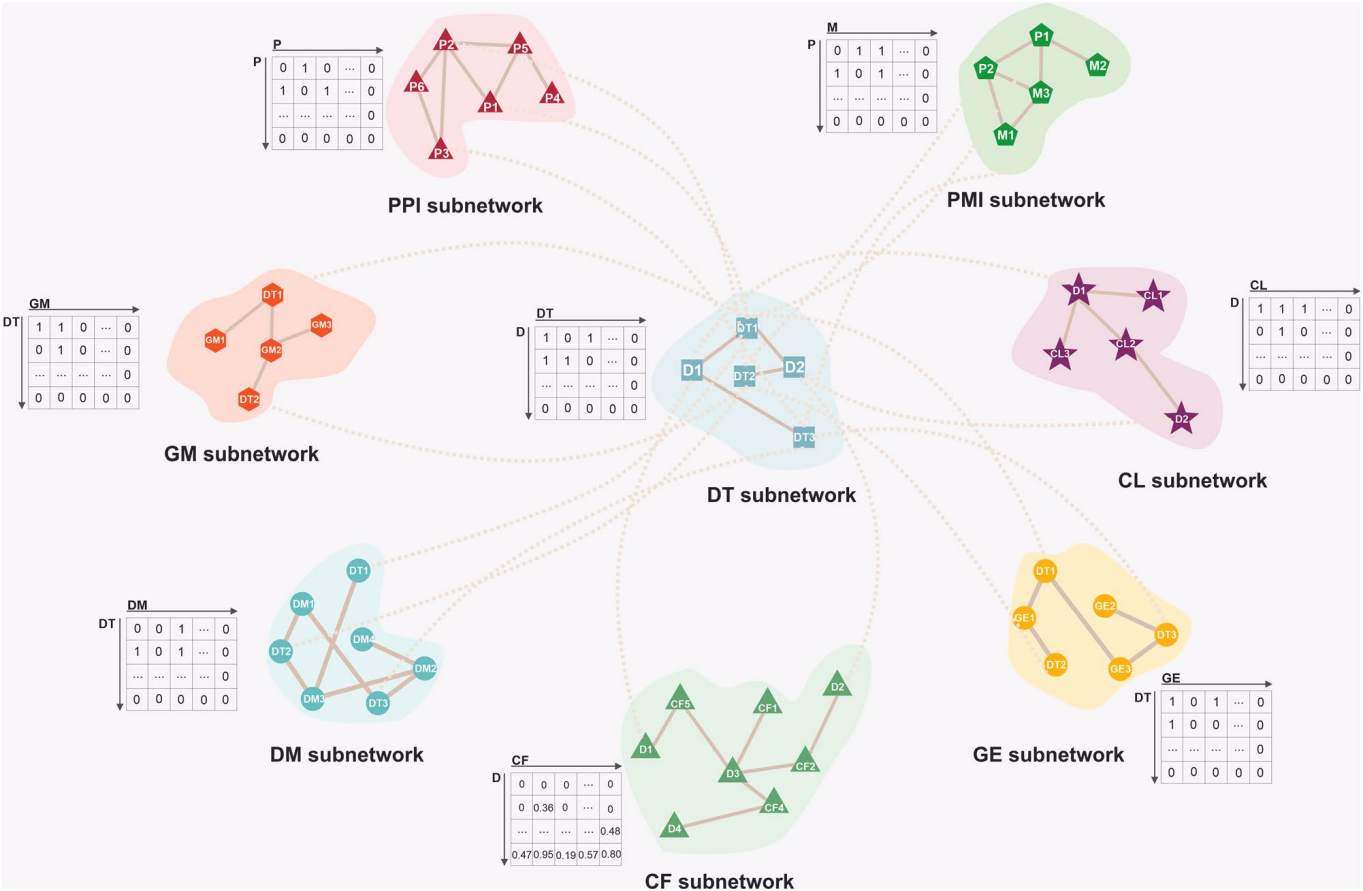
Construction of the heterogeneous network. The heterogeneous feature matrix was generated from drug-target, protein–protein interaction, protein-metabolite interaction, gene mutation, gene expression, differential methylation, chemical structures, and cell lines. The matrix values comprised zero, and non-zero values.

In this study, different types of features were utilized; hence we constructed an undirected network U = (V, E), where V is a set of N nodes, in which V = {V ^DT^ $\cup$ V ^PPI^ $\cup$ V ^PMI^ $\cup$ V ^GM^ $\cup$ V ^GE^ $\cup$ V ^DM^ $\cup$ V ^CF^ $\cup$ V ^CL^} is composed of cell lines, and seven sets of feature components, and E is a set of M edges such as protein–protein interaction and drug target. These N nodes have node feature vectors ${a}_{1},{a}_{2,}{a}_{3},\dots , {a}_{N} \in {\mathbb{R}}^{d}$ where d is the dimension of the feature vector. As for the edges, for example, ${(v}_{i},{v}_{j})$ represents the link between node ${v}_{i}$ and ${v}_{j}$. The total set of feature vectors has been combined to generate a feature matrix. As shown in Fig. 4, was defined feature matrix F as:

$$F=\left\{ \begin{array}{c} {F}^{D-DT}\in {\mathbb{R}}^{{N}_{{V}^{D}}\times {N}_{{V}^{DT}}}\\ if\,{v}_{i}\in {V}^{D}\text{,} \, {v}_{j} \in {V}^{DT\cdot }\\ {F}^{DT-PPI}\in {\mathbb{R}}^{{N}_{{V}^{DT}}\times {N}_{{V}^{PPI}}}\\ if\,{v}_{i} \in {V}^{DT}.{v}_{j} \in {V}^{PPI\cdot }\\ {F}^{DT-PMI}\in {\mathbb{R}}^{{N}_{{V}^{DT}}{\times N}_{{V}^{PMI}}}\\ if\,{v}_{i }\in {V}^{DT}.{ v}_{j}\in {V}^{PMI\cdot }\\ {F}^{DT-GM}\in {\mathbb{R}}^{{N}_{{V}^{DT}}\times {N}_{{V}^{GM}}}\\ if\,{v}_{i}\in {V}^{DT}.{v}_{j}\in {V}^{GM\cdot }\\ {F}^{DT-GE}\in {\mathbb{R}}^{{N}_{{V}^{DT}}\times {N}_{{V}^{GE}}} \\ if\,{v}_{i}\in {V}^{DT}.{v}_{j}\in {V}^{GE\cdot }\\ {F}^{DT-DM}\in {\mathbb{R}}^{{N}_{{V}^{DT}}\times {N}_{{V}^{DM}}}\\ if\,{v}_{i}\in {V}^{DT}.{v}_{j}\in {V}^{DM\cdot }\\ {F}^{D-CF}\in {\mathbb{R}}^{{N}_{{V}^{D}}\times {N}_{{V}^{CF}}}\\ if\,{v}_{i}\in {V}^{D}.{v}_{j}\in {V}^{CF\cdot }\\ {F}^{D-CL}\in {\mathbb{R}}^{{N}_{{V}^{D}}\times {N}_{{V}^{CL}}}\\ if\,{v}_{i}\in {V}^{D}.{v}_{j}\in {V}^{CL\cdot }\end{array}\right.$$

where D is a set of drugs that are related to cancer drugs. Hence, F_ij_ ∈ F^D−DT^ = 1 if a D node v_i_ and a DT node v_j_ are related according to the drug and drug target, F_ij_ = 0 otherwise, which means between drug and drug target there is no interaction. F^DT−PPI^ ∈ [0, n] is indicated the similarity values between pairs of PPI nodes, in which n represents the non-zero values. F^DT−PMI^ ∈ [0, n] is represented the non-zero values of similarity between pairs of PMI nodes. F_ij_ ∈ F^DT−GM^ = 1 indicates DT *i* is related according to the drug target and gene mutation with j-th GM, F_ij_ = 0 otherwise. F_ij_ ∈ F^DT−GE^ = 1 represents the i-th DT is associated with a j-th GE, F_ij_ = 0 otherwise. F_ij_ ∈ F^DT−DM^ = 1 if the interaction between a DT and DM nodes has been observed, F_ij_ = 0 otherwise. In this study, to represent the chemical features of drugs F^D−CF^ ∈ [0, n] is utilized. where D − CF is the chemical features of drugs, and n indicates the non-zero values of features. F_ij_ ∈ F^D−CL^ = 1 if a D node v_i_ and a CL node v_j_ are related according to the NCI-ALMANAC data, F_ij_ = 0 otherwise.

**Figure 5:**
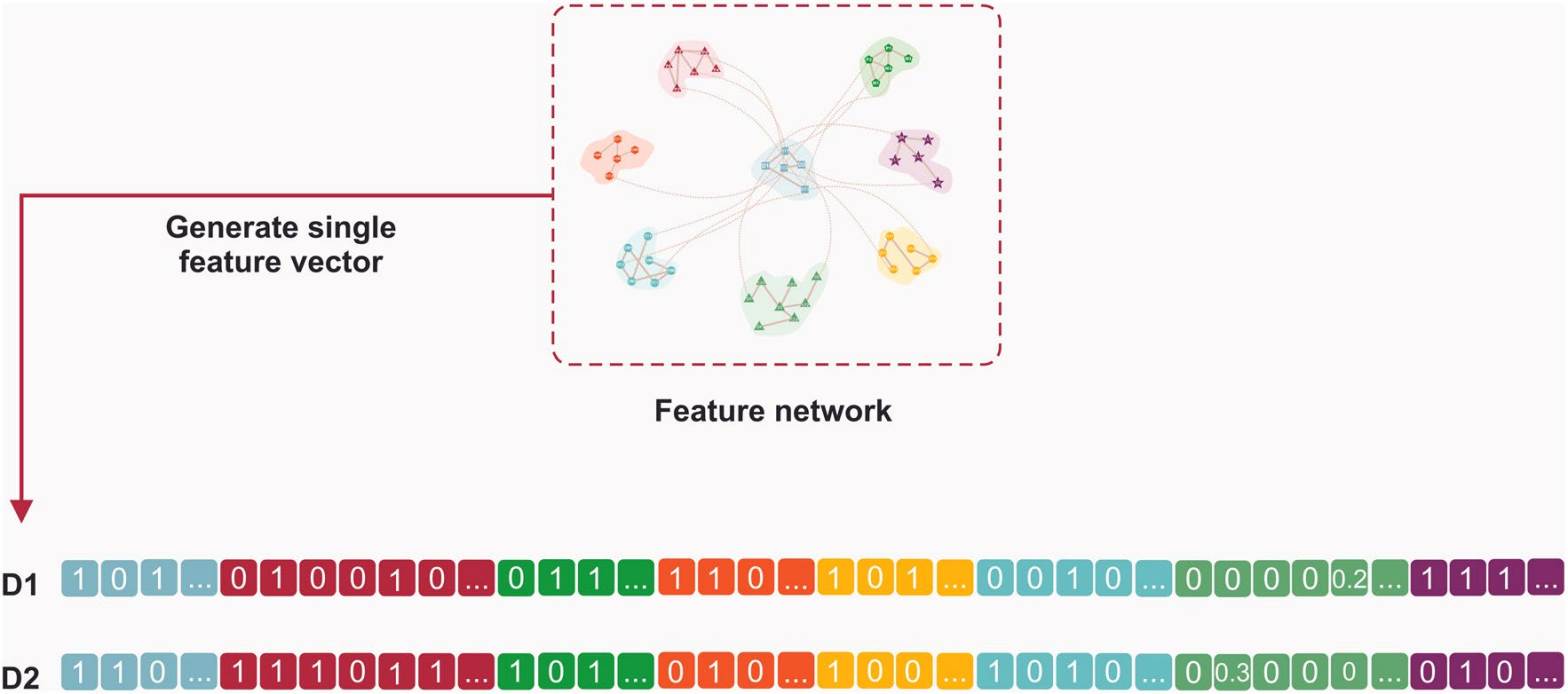
Single input feature vectors. Single input feature vectors derived from multi-topological structural relations among the drug-target, protein–protein interaction, protein-metabolite interaction, gene mutation, gene expression, differential methylation, chemical structures, and cell lines.

To utilize the topological structural relations among the cell lines, and seven sets of features embedded in the network U, we formulated the multi-structure of topology as single input feature vectors (Fig. 5). As an input feature vector, the vector was divided into eight different groups: seven sets of features, and cell line related features. As mentioned above, the value of each group consists of two sets: (i) zero, and (ii) non-zero values.

### Feature groups construction

The various features group was established to assess the benefits of features on classifying the drug synergy, along with the network of features. Six groups of features were constructed. Where, the first group consisted of drug targets and cell lines, and the last group was the network of features comprised of total feature groups. Table 3 details the information on groups.

**Table 3:** Feature group description.

Group	Group name	Features	Number of features
Group 1	DT_CL	Drug-target, Cell-lines	393
Group 2	DCGM	Drug-target, Cell-lines, Gene-mutation	488
Group 3	DCGME	Drug-target, Cell-lines, Gene-mutation, Gene-Expression	752
Group 4	DCGMEM	Drug-target, Cell-lines, Gene-mutation, Gene-Expression, Differential-methylation	1086
Group 5	DC_networkI	Drug-target, Cell-lines, Gene-mutation, Gene-Expression, Differential-methylation, Chemical-features	1612
Group 6	DC_networkII	Drug-target, Cell-lines, Gene-mutation, Gene-Expression, Differential-methylation, Chemical-features, Protein–Protein Interaction, Protein-metabolite Interaction	1614

The name of each group has been determined based on their feature groups.

*DT_CL* drug targets and cell lines, *DCGM* drug targets, cell lines, and gene mutation, *DCGME* drug targets, cell lines, gene mutation, and gene expression, *DCGMEM* drug targets, cell lines, gene mutation, gene expression, and differential methylation, *DC_networkI* drug targets, cell lines, gene mutation, gene expression, differential methylation, and chemical features, *DC_networkII* drug targets, cell lines, gene mutation, gene expression, differential methylation, chemical features, protein–protein interaction, protein metabolite interaction.

### Construction of deep neural network model

**Figure 6:**
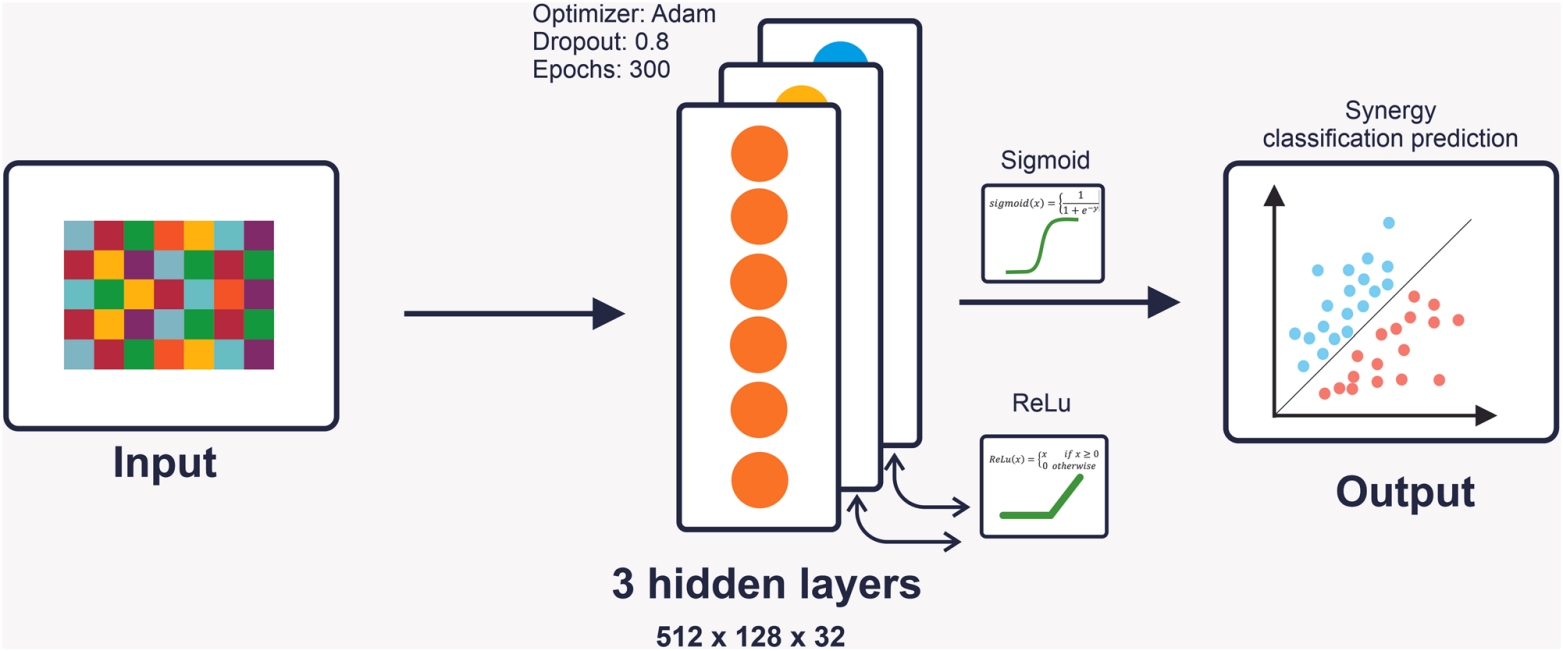
Schematic illustration of the proposed method (SYNDEEP). The model takes the feature vectors as input. The input layer is connected to 3 hidden layers. The number of nodes in each of the three hidden layers was 512, 128, and 32, respectively. Between each of the three hidden layers, there is a ReLu activation function. For the last layer, we adopt a sigmoid activation function. The learning rate was 10^−2^.

The Multi‑layer perceptron (MLP) was selected to build the model for synergy prediction. The shape of the MLP model was conic. The different hyperparameter settings were considered (i.e. number of layers, number of neurons, and the learning rate). The six diverse different layers (3, 4, 5, 6, 7) were tested. Various numbers of neurons (1024, 512, 256, 128, 64, 32) and learning rates (10^−1^, 10^−2^, 10^−5^, 10^−8^, 10^−75^) were examined. After comparing the outcomes of different deep networks, the best result was observed from the five-layer network for binary classification of the drug synergy. The selected model had an input layer, 3 hidden layers, and an output layer (Fig. 6). The two activation functions have been used in the model: the ReLu and the sigmoid functions.

$$ReLu\left(x\right)=\left\{\begin{array}{c}x\,if\,x\ge 0\\ 0 \,otherwise \end{array}\right.$$

The ReLu activation function is termed Rectified Linear Unit or rectifier, which is one of the common activation functions for deep learning. The ReLu function is used in hidden layers to detect the patterns, and the performance of the model increase by overcoming the gradient vanishing. The activation function of the last layer was the sigmoid function:

$$sigmoid\left(x\right)=\left\{\frac{1}{1+{e}^{-y}}\right.$$

To optimize, we used the binary cross-entropy function as a loss function.

$$L= -\frac{1}{N} \sum_{i=1}^{N}{y}_{i}\cdot \mathrm{log}\left({s}_{i}\right)+\left(1-{y}_{i}\right)\cdot \mathrm{log}\left(1-{s}_{i}\right)$$

where y_i_ is the actual synergy label of each drug pair, s_i_ is the predicted synergy score of each combination, and N is the number of drug combinations.

### Applying other machine learning methods

To evaluate the performance of the predictive model, we compared the deep neural network model with different machine learning algorithms. Shallow neural network (SNN), k-nearest neighbor (KNN), Support vector machines (SVMs), random forest (RF), and gradient boosting classifiers (GBCs) were the algorithms that were recruited in this step. All the methods were examined by different hyperparameters. The performance of considered algorithms is largely dependent on hyperparameters. Therefore, the various hyperparameters of the algorithms were adjusted to the optimal state.

### Evaluation criteria of presented models

The tenfold cross-validation and some popular evaluation criteria were utilized in the proposed experiments to evaluate the models’ performance. The tenfold cross-validation involves randomly splitting the whole dataset into ten independent subsets equally sized. Each time one fold is used for testing, and nine remaining folds are used for the training, which this procedure is repeated iteratively. This process is performed ten times to ensure each fold is tested at once. To evaluate the performance of the prediction model, widely used evaluation criteria, including accuracy (Accu.), sensitivity (Sen.), specificity (Spec.), precision (Prec.), F-Score (F_score_), Matthews Correlation Coefficient (MCC.), and Cohen's kappa coefficient (Kappa) were calculated as follow.

$$Accu. = \frac{TP+TN}{TP+TN+FP+FN}$$

$$Sen. = \frac{TP}{TP+FN}$$

$$Spec. = \frac{TN}{TN+FP}$$

$$Prec. = \frac{TP}{TP+FP}$$

$${F}_{score}=2\times \frac{Sen. \times Prec. }{Sen. +Prec. }$$

$$MCC. = \frac{TP\times TN-FP\times FN}{\sqrt{\left(TP+FP\right)\left(TP+FN\right)\left(TN+FP\right)\left(TN+FN\right)}}$$

$$Kappa. = \frac{Accu. -{P}_{c}}{1-{P}_{c}}$$

where TP, FP, TN, and FN denote true positive, false positive, true negative, and false negative, respectively. The current study used Cohen’s kappa to evaluate the agreement grade between observed accuracy and expected accuracy and the performance of the prediction model. Where P_c_ is the probability value of agreements expected by chance.

In addition, we performed McNemar’s Test to compare SYNDEEP results with proposed state-of-the-art computational models. McNemar’s Test is defined as follows:

$${X}^{2} = \frac{{(\left|B-C\right|-1)}^{2}}{B+C}$$

where B is the number of drug combinations that were detected correctly as non-synergy and incorrectly detected as synergy effects, while C is the number of drug combinations that were detected correctly as synergy and incorrectly as non-synergy effects.

### Computational equipment

In this study, the deep neural network model is implemented in python language using Python 3.7 version. To implement the deep learning methods and machine learning algorithms, we used the Keras and Scikit-learn libraries, respectively. The software environment to develop the experiments was Google colab.

## Data Availability

Dataset: NCI-ALMANAC Data Resource (https://dtp.cancer.gov/ncialmanac). Drug-target Interactions Data: Drug-target interactions (https://go.drugbank.com/releases/latest). Protein–Protein Interactions Data: PPI (https://string-db.org/). Genomic Features Data: gene expression, mutations, and differential methylation (https://cancer.sanger.ac.uk/cosmic). Protein-Metabolite Interaction Data: protein-metabolite interaction (https://www.mhmdb.co.uk/). Scripts: The source code of SYNDEEP is available in (https://github.com/annatorkamannia/SYNDEEP).
